# Familiarization with treadmill walking: How much is enough?

**DOI:** 10.1038/s41598-019-41721-0

**Published:** 2019-03-26

**Authors:** Christian Meyer, Tim Killeen, Christopher S. Easthope, Armin Curt, Marc Bolliger, Michael Linnebank, Björn Zörner, Linard Filli

**Affiliations:** 1 Spinal Cord Injury Center, Balgrist University Hospital, Forchstrasse 340, Zurich, Switzerland; 2 Department of Neurology, University Hospital and University of Zurich, Frauenklinikstrasse 26, Zurich, Switzerland; 3 Department of Neurology, Helios-Klinik Hagen-Ambrock, Witten/Herdecke University, Ambrocker Weg 60, 58091 Hagen, Germany

## Abstract

Treadmill-based gait analysis is widely used to investigate walking pathologies and quantify treatment effects on locomotion. Differential sensorimotor conditions during overground vs. treadmill walking necessitate initial familiarization to treadmill walking. Currently, there is no standardized treadmill acclimatization protocol and insufficient familiarization potentially confounds analyses. We monitored initial adaptations to treadmill walking in 40 healthy adults. Twenty-six walking parameters were assessed over 10 minutes with marker-based kinematic analysis and acclimatization profiles were generated. While 16 walking parameters demonstrated initial acclimatization followed by plateau performance, ten parameters remained stable. Distal lower limb control including ankle range of motion, toe trajectory and foot clearance underwent substantial adaptations. Moreover, intralimb coordination and gait variability also demonstrated acclimatization, while measures of symmetry and interlimb coordination did not. All parameters exhibiting a plateau after acclimatization did so within 6–7 minutes (425 strides). Older participants and those naïve to treadmill walking showed adaptations with higher amplitudes but over similar timescales. Our results suggest a minimum of 6 minutes treadmill acclimatization is required to reach a stable performance, and that this should suffice for both older and naïve healthy adults. The presented data aids in optimizing treadmill-based gait analysis and contributes to improving locomotor assessments in research and clinical settings.

## Introduction

Gait disorders are commonly encountered in patients with orthopedic and neurological disorders and in otherwise healthy aging^1–3^. Walking impairment is associated with significant reductions in functional independence and an increased risk of falls and subsequent injury, morbidity and mortality^4–6^. Investigations into new therapies and interventions for locomotor disorders generally use ordinal clinical scores and scales, often with poor sensitivity and reliability, as well as simple time- or distance-based walking assessments to measure therapeutic responses^7–9^.

Instrumented gait analysis offers distinct advantages over such functional walking assessments as direct inferences regarding the mechanism of deficits and treatment responses can be made by analyzing kinetic and kinematic parameters^10–12^. Such analyses may be performed during overground walking, but this needs large acquisition volumes and invariably requires participants to execute turns or walk in circular paths, complicating data processing and interpretation. Treadmill-based gait analysis can be undertaken in relatively small laboratories or in clinical settings and allows for the collection of many consecutive gait cycles and precise control of gait velocity^13–15^. To what extent analyses of treadmill walking can be generalized to everyday walking is debated^16,17^, but acclimatization to the often unfamiliar task of walking on a moving belt is known to mitigate some treadmill-related differences^17–20^. While several studies have demonstrated a highly comparable walking pattern during overground and treadmill walking after sufficient acclimatization to the latter^18,19,21,22^, there is currently no consensus on how much acclimatization is needed.

Studies investigating the acclimatization period to treadmill walking in young, healthy individuals have reported divergent findings ranging from a few seconds to many minutes^19,22–27^. Wall & Charteris investigated angular and temporal aspects of gait and found that a large degree of acclimatization occurs in the first 10 seconds of treadmill walking, but that a true steady-state is not reached even after 10 minutes^23^. Later work reported that most spatio-temporal parameters reached steady-state after around 30 seconds of treadmill walking, although stride length required 10 minutes to reach a plateau^24^. Three studies with different kinematic and kinetic outcome measures have similarly reported acclimatization periods between 4 to 6 minutes^22,25,26^. While Wass, Taylor, & Matsas reported that older individuals were not able to familiarize to the treadmill even after 15 minutes^27^, Schellenbach *et al*. found that older subjects showed only minor changes in gait pattern after the same period of 15 minutes of treadmill walking in a virtual environment and that this period was comparable to that required by young participants^19^. Older subjects did, however, show a tendency towards higher amplitudes of acclimatization, especially for the parameters step length and cadence. The contradictory results on acclimatization periods may be partly explained by the studies’ use of different definitions of acclimatization or by divergent outcome measures, as different parameters may be associated with different acclimatization durations. These studies also differed in whether the use of handrails was permitted during treadmill walking. Furthermore, the potential confounding factor of treadmill experience has rarely been reported. Detailed knowledge of acclimatization periods and the mechanisms underlying familiarization to treadmill walking would inform assessment protocols and facilitate interpretation of treadmill-based gait analysis in clinical and research settings.

The aim of this study was to investigate the process of acclimatization to treadmill walking in healthy individuals and to determine the duration of the process, looking specifically at the evolution of 26 key gait parameters.

## Material and Methods

This study was approved by the Zurich cantonal ethics committee (KEK-2013-0601). All experiments were performed at the University Hospital Zurich in accordance with the standards of the Declaration of Helsinki and Good Clinical Practice guidelines. Participants were recruited locally via flyers and posters into two predefined age groups; 21–50 years and 51–80 years, with 20 participants and an equal gender distribution in each group. Participants were excluded if they reported any treadmill experience within the last 60 days or if any orthopedic or neurological diagnoses were reported. All participants gave written, informed consent.

Participants underwent a detailed clinical examination by a physician to exclude neurological or locomotor pathology before their maximum walking velocity was determined using the mean of two trials of the timed 25-foot walk test (T25FW)^28^. All experiments were performed in a dedicated gait laboratory equipped with an instrumented treadmill (FDM-T, zebris medical GmbH, Germany). The treadmill was equipped with handrails and a transparent, non-weight-bearing security harness. All participants were instructed to use the handrails only if required to prevent a fall. A 3D motion capture system (Vicon, UK) with 14 infrared cameras (Vicon Bonita) was used for data collection at a sampling frequency of 200 Hz. Forty-six, 14 mm-diameter spherical reflective markers were fixed directly on the skin over anatomical landmarks according to a modified Plug-In-Gait marker constellation^29^. During all recordings, participants were asked to fix their gaze on a black cross displayed on a 22-inch monitor placed at eye-height in front of the treadmill.

Participants began walking on the treadmill at a constant velocity of 50% of their maximum overground walking velocity, without acclimatization. The objective speed definition (50% of maximal walking speed) allows to assess gait function at a walking speed that is proportional to participants’ walking ability. The treadmill was accelerated quickly (1.2 m/s^2^) and data collection was started manually as soon as the treadmill reached the target velocity. Kinematic recording continued while the participant walked for 10 minutes. Participants were asked to indicate when they felt fully adapted to the treadmill by saying “yes”. Besides this, any interaction with or disturbance of the participant during recording was not permitted.

Data were processed using Vicon Nexus 2.2.3 motion capture software (Vicon, Oxford, UK). Proprietary algorithms within the software were used to fill gaps in the marker trajectories before a low-pass Woltring filter was applied to all trajectories. The position of the hip joint rotation center was calculated using the Plug-in-Gait model; all other calculations were performed using a 3D vector-based approach without further modelling^30,31^. Parameters of interest were extracted using ProCalc 1.1 (Vicon, Oxford, UK) gait analysis software and temporal gait events were defined by velocity zero-crossings of the toe and heel markers as described previously^32,33^. Twenty-six outcome parameters of interest were calculated for each step cycle and extracted in 25-step bins using ProCalc and Matlab (2013a & 2017a, Mathworks Inc. Natick, MA, USA). The following parameters were calculated and classified into eight functional domains:

1. *End-point measures*: step length, toe height, toe trajectory length;
2. *Range of motion (ROM)*: hip ROM, knee ROM, ankle ROM, calculated as angles between vectors;
3. *Stability*: step width, C7 trajectory length, center of mass (COM) path medio-lateral, COM path antero-posterior;
4. *Symmetry*: ratio (left/right) of hip ROM, knee ROM, ankle ROM;
5. *Interlimb coordination*: temporal coordination of right and left extremities as defined by phase dispersion^34,35^;
6. *Intralimb coordination*: shape differences of hip-knee and knee-ankle cyclograms were characterized as the square root of the sum of squared distances (SSD^36^) with the last bin as reference cyclogram, and cycle to cycle consistency of the hip-knee and knee-ankle cyclograms within a bin was quantified by the angular component of coefficient of correspondence (ACC^37^);
7. *Gait phases*: double limb support phase (DLS), swing phase, stride time;
8. *Variability*: coefficient of variation (COV) within a bin of step length, C7 trajectory length and stride time.

Except for symmetry parameters, bilateral parameters were only considered on the right side.

### Data analysis & Statistics

For each parameter, mean and COV were calculated for each bin of 25 strides for each individual and each group. All participants achieved at least 20 full bins over 10 minutes, so each contributed their first 20 bins to the analysis. Kolmogorov-Smirnov and Shapiro-Wilk tests indicated that most parameters were not normally distributed within bins. Consequently, non-parametric statistical tests were used for statistical analyses at a significance level of α = 0.05.

To describe the process of acclimatization, the first bin (representing initial performance) was tested against all later bins (performance at later time points) using repeated measures Friedman tests followed by corrected Dunn’s post-hoc tests.

To characterize the acclimatization process of a given parameter, we defined 4 categories of acclimatization *a priori*. These categories of acclimatization cover the principal and most frequent forms of gait adaptations during familiarization to treadmill walking in healthy subjects. We characterized the 4 categories using statistical criteria, without any presumptions to the course or direction of a parameter’s evolution:

1. Acclimatization with plateau: ≥4 consecutive bins must be statistically significantly different from the first bin in post-hoc tests for plateau onset to be defined. The plateau is maintained if no subsequent bin is significantly different from the initial bin of the plateau (if this requirement is not met, plateau onset is moved to a later bin until criteria are met), and no run of ≥4 bins is no longer significantly different from the first bin.
2. Steady acclimatization without plateau: Plateau onset as defined in 1 but subsequent bins after plateau onset are significantly different from the initial bin of the plateau.
3. Acclimatization with interim plateau: a plateau as defined in (1) ends if ≥4 further consecutive bins were no longer significantly different from the first bin. No further plateaus meeting criteria (1) are reached.
4. No acclimatization: none of the above criteria apply.

The intention of categories 1 and 3 was the detection of plateaus and the exclusion of fluctuations. Parameters showing no acclimatization, either in the form of no significant changes from baseline (category 4) or exhibiting a steady and unidirectional change (category 2), indicating incomplete familiarization within the 10 minutes, were also captured by our classification algorithm. The temporal onset of a plateau is reported with the mean number of steps taken to reach steady-state and the corresponding time is calculated using the mean stride time of all participants.

To characterize the acclimatization of locomotion in more detail, the amplitude and the direction of changes were determined for every gait parameter. For this purpose, the absolute and relative change of every bin with respect to the first bin were calculated for the group. The maximum change was used as a measure for the amplitude and the direction of acclimatization for those parameters meeting the acclimatization criteria.

### Subgroup analyses

To investigate the effects of the factors age, gender and treadmill experience (no treadmill experience; treadmill experience >12 months; <12 months), participants were separated into subgroups for each covariate. Acclimatization definitions were the same for these subgroups as the overall cohort. Subgroup differences in frequency of parameters with acclimatization were analyzed by Fisher’s exact test.

## Results

**Figure 1:**
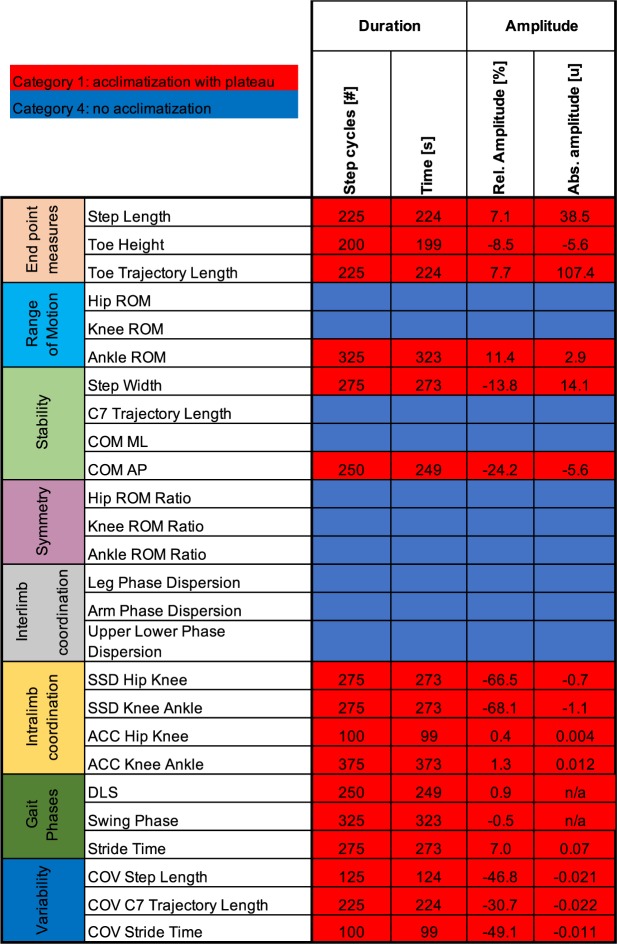
Acclimatization profiles of 26 key gait parameters during treadmill walking. Twenty-six key gait parameters were assessed with respect to acclimatization periods and amplitudes during treadmill walking. Stable parameters without any acclimatization are marked in blue (category 4, see methods), whereas walking parameters demonstrating acclimatization resulting in stable plateau performance are illustrated in red (category 1). The duration of acclimatization (i.e. time to reach a stable plateau) is indicated in number of strides and seconds. Relative and absolute amplitudes of adaptations are provided, indicating the degree and direction of acclimatization. Absolute amplitudes are given in the following units: step length [mm], toe height [mm], toe trajectory length [mm], Ankle ROM [deg], Step width [mm], COM AP [mm], SSD Hip Knee [au], SSD Knee Ankle [au], ACC Hip Knee [au], ACC Knee Ankle [au], Stride time [s], COV step length [au], COV C7 trajectory length [au], and COV stride time [au]. For the parameters with % as unit (DLS, swing phase), relative amplitudes represent the absolute difference to the first bin (i.e. absolute differences do not exist for these parameters). Abbreviations: Abs.: absolute; au: arbitrary units; ACC: angular component of coefficient of correspondence; COM: center of mass; COV: coefficient of variation; DLS: double limb support; ROM: range of motion; Rel.: relative; SSD: sum of squared distances.

**Figure 2:**
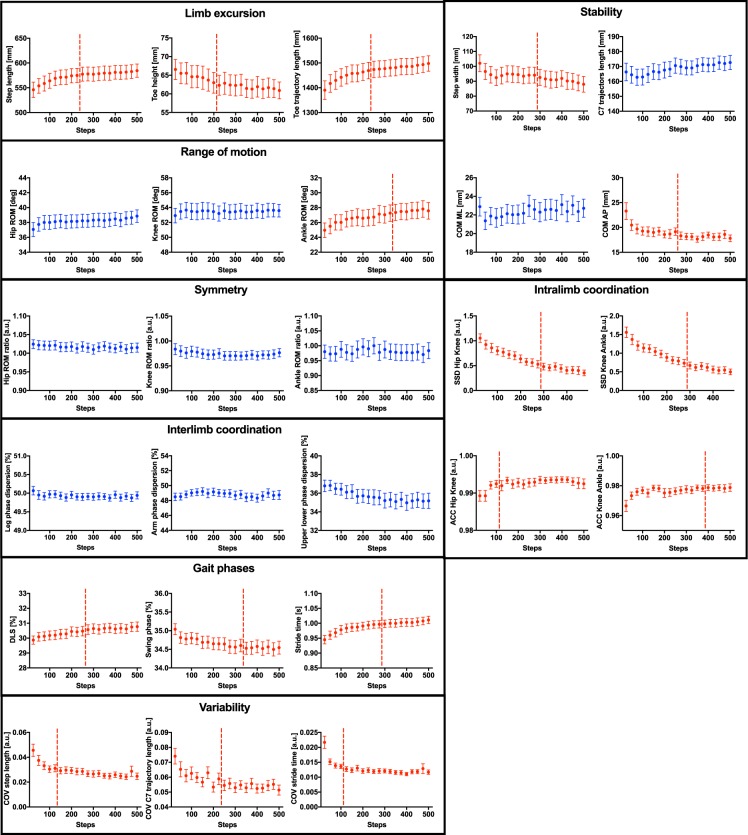
Adaptation curves of all locomotor parameters during acclimatization to treadmill walking. Twenty-six locomotor parameters were monitored in 40 healthy subjects over the course of 10 minutes of treadmill walking (i.e. 20 bins of 25 strides each). Data represent mean values ± SEM. Stable parameters without acclimatization according to our pre-defined criteria (category 4, see methods) are shown in blue, whereas parameters revealing acclimatization (category 1) are highlighted in red. Plateau onset (if applicable; category 1) is illustrated by a horizontal red dashed line. Abbreviations: ACC: angular component of coefficient of correspondence; COM: center of mass; COV: coefficient of variation; DLS: double limb support; ROM: range of motion; SSD: sum of squared distances.

Forty healthy subjects were included in this study (age [mean ± standard deviation]: 51.0 ± 17.6 years; height: 170.3 ± 11.0 cm; weight: 67.7 ± 12.9 kg; 20 females). Mean treadmill speed (±standard deviation) was 4.3 ± 0.6 km/h. All participants were able to walk unassisted (i.e. without using the handrails). Considerable adaptations of the gait pattern were observed during initial treadmill walking, but not all 26 parameters studied were affected (Fig. 1). Ten parameters were stable throughout recording (category 4), whereas 16 parameters showed significant adaptations resulting in a stable performance only after a certain acclimatization period (category 1; Figs 1 and 2). The duration of this acclimatization period ranged from 100 to 375 strides (mean = 239 strides) depending on the parameter investigated, corresponding to a time of 99 to 373 seconds (mean = 237 s). The shortest acclimatization period was exhibited by variability parameters (ACC hip-knee, COV step length, COV stride time). Generally, acclimatization periods were longer for the more distal (i.e. ankle ROM and AAC knee-ankle) versus proximal aspects of gait (i.e. hip ROM, knee ROM, ACC hip-knee). The subjective rating of the onset of stable performance was 95 ± 120 seconds, shorter than the mean objective quantification for any gait parameters. No category 2 or 3 acclimatization profiles (i.e. steady acclimatization or fluctuations) were observed in the overall cohort (Fig. 1).

**Figure 3:**
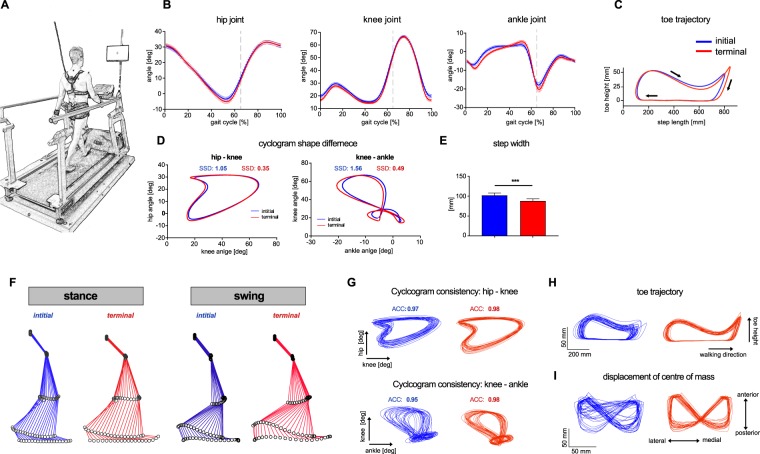
Adaptations of the locomotor pattern during familiarization with treadmill walking. (**A**) Illustration of the experimental setup. (**B**–**I**) Representation of initial (fist bin; blue) and terminal (last bin; red) treadmill walking performance. (**B**) Mean sagittal excursions of the hip, knee and ankle joint (n = 40). Data represent mean values ± SEM. (**C**) Mean toe trajectory (n = 40). (**D**) Mean hip-knee and knee-ankle cyclograms (n = 40). (**E**) Step width (mean ± SEM, n = 40, Wilcoxon signed-rank test, p < 0.0001). (**F**) Stick figures representing leg movements during stance and swing phase of a representative subject. (**G**) Hip-knee and knee- ankle cyclograms of 25 strides of a representative subject. (**H**) Toe trajectories of 25 strides for a representative subject. (**I**) 2D-representation of the COM displacement on the floor for 25 strides of a representative subject. Representative data in (**F**–**I**) were collected from the same participant. Abbreviations: ACC: angular component of coefficient of correspondence; SEM: standard error of the mean; SSD: sum of squared distances.

All parameters in the locomotor domains of *end point measures*, *intralimb coordination*, *gait phases* and *variability* showed significant adaptations to treadmill walking (Figs 1 and 2). Some parameters within the functional domains *range of motion* (1/3) and *stability* (2/4) showed evidence of acclimatization within 10 minutes, while no parameters relating to *symmetry* or *interlimb coordination* did so. Parameters characterizing *end point measures* showed relative changes due to acclimatization of about 8%, exemplified in the study population by increased step length and toe trajectory length, as well as by a reduced mid-swing toe height (Fig. 3C). Step-to-step consistency improved considerably after initial acclimatization as indicated by reduced COV values (Fig. 1) and enhanced consistency of angle-angle cyclograms and toe trajectory (Fig. 3G,H). Acclimatization-driven changes in leg joint movements (ROM) were only observed at the ankle joint, which displayed increased excursions corresponding to improved foot roll dynamics (higher dorsal extension prior to toe off; higher plantarflexion at toe off and after heelstrike) after initial familiarization (Fig. 3B). Hip and knee excursions exhibited only minor adaptations, in keeping with the lack of significant acclimatization for the ROM of these joints (Fig. 3B). Adaptations in the *stability* domain resulted not only in decreased step width (Fig. 3E) and anterior-posterior excursion of the COM for the whole group but were also observable as improved consistency of the COM trajectory at an individual level (Fig. 3I).

**Figure 4:**
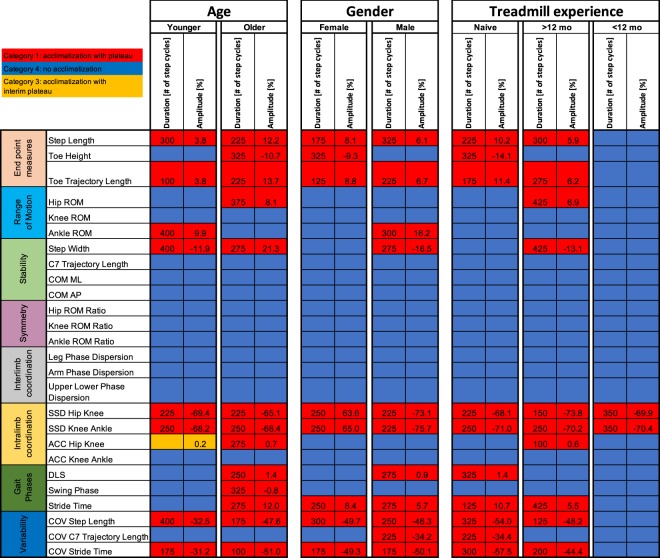
Effects of covariates age, gender and treadmill experience on acclimatization profiles during treadmill walking. Acclimatization of the twenty-six key walking parameters was characterized in three subgroup analyses. Stable parameters without any acclimatization are marked for each subgroup in blue (category 4, see methods), whereas walking parameters revealing acclimatization resulting in a stable plateau performance are illustrated in red (category 1). The duration of acclimatization (i.e. time to reach a stable plateau) is indicated for all subgroups in number of strides and seconds. Relative and absolute amplitudes of adaptations are provided to assess the extent of acclimatization. Reltative amplitudes (highest difference to first bin) of acclimatization are provided in percent. For the parameters with % as unit (DLS, swing phase), relative amplitudes represent the absolute difference to the first bin (i.e. absolute differences do not exist for these parameters). Abbreviations: Abs.: absolute; au: arbitrary units; ACC: angular component of coefficient of correspondence; COM: centre of mass; COV: coefficient of variation; DLS: double limb support; ROM: range of motion; Rel.: relative; SSD: sum of squared distances.

In a subgroup analysis, we evaluated the effects of the covariates age, gender and treadmill experience on acclimatization of the gait pattern during initial treadmill walking. Detailed characteristics of the subgroups are provided in Table 1. The younger and older age groups were demographically similar, however, older subjects walked significantly slower (4.7 ± 0.5 vs 4.0 ± 0.6 km/h; Table 1). Overall, the number of gait parameters showing significant adaptations during treadmill walking was not different between younger and older participants (Fig. 4; p = 0.40; Fisher’s exact test). One category 3 acclimatization with interim plateau was observed in the younger subgroup (ACC Hip Knee; Table 1). Relative adaptation amplitudes were generally higher in the older group, with, for example, step length adaptation 3.8% in younger and 12.2% in older adults. Likewise, adaptation of stride time variability (COV stride time) was −31.2% in the younger group vs. −51.0% in the older group. In contrast, the *duration* of the acclimatization to treadmill walking was comparable between groups (*younger:* range = 100–400 strides, median = 275 strides; *older:* range = 100–375 strides, median = 250 strides).

**Table 1:** Demographic characteristics of subgroups defined based on age, gender and treadmill experience.

	Younger	Older	P-Value	
Volunteers [n]	20	20	na	
Age [y]	35.5 ± 8.6	66.5 ± 7.7	<0.0001	
Height [cm]	172.6 ± 10.9	168.1 ± 11.0	0.1963	
Weight [kg]	67.3 ± 14.0	68.2 ± 12.1	0.8384	
Treadmill Speed [km/h]	4.7 ± 0.5	4.0 ± 0.6	0.0003	
Female [%]	50	50	na	
	**Male**	**Female**	**P-Value**	
Volunteers [n]	20	20	na	
Age [y]	50.6 ± 18.8	51.5 ± 16.8	0.862	
Height [cm]	178.5 ± 8.5	162.2 ± 6.0	<0.0001	
Weight [kg]	76.2 ± 11.9	59.2 ± 7.1	<0.0001	
Treadmill Speed [km/h]	4.4 ± 0.6	4.2 ± 0.6	0.355	
Female [%]	na	na	na	
	**Naive**	**>12 mo**	**<12 mo**	**P-Value**
Volunteers [n]	15	16	9	na
Age [y]	57.7 ± 15.8	48.5 ± 17.3	44.3 ± 19.4	0.112
Height [cm]	169.5 ± 12.5	170.6 ± 9.9	171.3 ± 11.6	0.921
Weight [kg]	69.3 ± 13.9	67.2 ± 12.6	66.1 ± 13.0	0.829
Treadmill Speed [km/h]	4.2 ± 0.8	4.4 ± 0.5	4.5 ± 0.4	0.548
Female [%]	40	50	66.7	0.449

Values represent participant numbers, the mean ± standard deviation or proportions (gender). P-values (right column of each table) represent results of the statistical subgroup comparisons (i.e. unpaired t-tests for the variables age,height, weight and treadmill speed; chi-square test for gender). Abbreviations: mo: months, na: not applicable.

Male and female participants showed significant differences in height and weight, but not in age nor treadmill speed (Table 1). Males demonstrated adaptations in a similar number of parameters as females (Fig. 4; 8 vs 11; *p* = *0*.*57*, Fisher’s exact test). Duration and amplitude of the adaptations were comparable between genders (*female:* range = 125–325 strides, median = 250 strides; *male:* range = 175–325 strides, median = 250 strides).

No significant difference in demographic characteristics or treadmill speed was observed between the subgroups defined by differential treadmill experience (Table 1). Subjects that had walked on a treadmill within the past 12 months showed the fewest parameters with significant adaptations (2 parameters) compared to those with treadmill experience >12 months ago (10 parameters) and entirely naïve treadmill walkers (10 parameters; over the 3 groups: *p* = *0*.*01;* Fisher’s exact test). More experience with treadmill walking was associated with smaller amplitudes of locomotor adaptation (naive vs. >12 months), whereas the duration of acclimatization was again similar between these subgroups (*naïve:* range = 125–325 strides, median = 237.5 strides; >12 months: range = 100–425 strides; median = 250 strides; <12 months: 350 strides). Subgroup comparison of amplitudes and duration of acclimatization with the most experienced group were not performed, as only two showed evidence of acclimatization.

## Discussion

We present comprehensive norm data for 26 key gait parameters with acclimatization periods and amplitudes in healthy adults during treadmill walking. A majority of parameters showed significant plateaus after an initial familiarization period, with adaptation amplitudes from the first steps on the treadmill to the plateau phase of up to 68%. Of those parameters reaching an acclimatization plateau, all did so within 6 minutes of treadmill walking, suggesting this may be the minimal necessary acclimatization duration for treadmill-based kinematic gait analysis. Self-perceived attainment of plateau performance substantially preceded the plateau onset as defined by objective locomotor assessments, speaking against simple subjective criteria for the identification of stable walking performance.

While an acclimatization period of 6 minutes is similar to that observed in some previous studies^22,25,26^, others reported shorter or longer periods^19,23,27^. In our study, different parameters were associated with quite different acclimatization patterns, likely accounting for the discrepancies and heterogeneity in the literature to date. Moreover, other studies used different measurement modalities (e.g. use of handrails, definition of plateaus, previous treadmill experience) and definitions of acclimatization, further hampering the comparison of findings between studies. Differences in acclimatization between gait parameters might be related to the fact that particular aspects of locomotion are controlled by different neural structures^38,39^. Several of the parameters that underwent considerable acclimatization were those for which cortical control and the integration/feedback of visual, vestibular and somatosensory inputs might be more important – specifically those in the variability domain and relating to distal endpoint control^40–43^. Those parameters which showed little modulation were related to symmetry, interlimb coordination and proximal ROM, aspects of gait thought to be more influenced by brainstem and spinal centers, including the spinal pattern generator^44–46^. Processing and integrating sensory feedback in the novel treadmill setting is therefore evidenced in the former, more supraspinally-controlled parameters exhibiting feedback-driven motor adaptation and learning^47,48^.

Treadmill and overground walking are different locomotor tasks and vary in terms of sensory feedback in particular. During treadmill walking, visual flow is absent^49^, somatosensory perception is altered due to the moving belt^13^ and vestibular demands differ due to lack of propagation acceleration. It remains unclear whether some or all of the adaptations seen here represent reversion of an untrained treadmill gait to one more representative of everyday, overground walking, or if the steady state reached is reflective of specifically-optimized locomotion in the unnatural environment of a motorized treadmill. Previous studies reported that spatiotemporal parameters are highly comparable between overground and treadmill walking^18,19,21,22^. However, decreased gait variability during treadmill walking compared to overground walking has been reported^50,51^, which might be compatible with the rapid decreases in the variability domain seen in our analysis. Overall, it may be assumed that many adaptations observed during treadmill walking likely lead to a more natural walking pattern resembling overground walking with the exception of gait variability parameters, where generalization to overground walking may require particular caution.

Covariate analysis of age, gender and treadmill experience revealed an influence of age and experience on familiarization with treadmill walking. Older individuals consistently demonstrated acclimatization amplitudes in the same direction but greater than their younger counterparts. The duration to plateau was broadly similar in both age groups, in keeping with previous work suggesting that older subjects begin walking on the treadmill with a gait pattern further from their optimal performance, perhaps due to increased anxiety regarding falling^52^, reduced sensory perception/integration^53^ or impaired motor learning/adaptive capacity^54^, but remain capable of adapting within the same timeframe as younger adults^19^.

Similarly, those naïve to treadmill walking also showed greater acclimatization amplitudes while exhibiting similar durations to those with experience of the modality. Treadmill experience appears to mitigate the degree to which a participant must adapt their walking pattern but not the duration that this adaptation requires. This suggests that the lower amplitude of acclimatization is a manifestation of retained motor learning, leading to a long-term change of behavior, whereas duration could represent motor adaptation to locomotor environment^47,48^.

Several limitations must be considered; firstly, only one, relative gait speed was assessed (50% of maximal overground walking speed; no handrail support), thus restricting the generalizability of our acclimatization profiles. The applied speed definition resulted in a moderate and sustainable speed that was perceived as comfortable by most participants. However, the speed might not necessarily correspond to preferred walking speed. Secondly, due to the bin sampling approach, the resolution of acclimatization effects is limited to 25 steps, although the functional relevance of fluctuations below this resolution is questionable. Furthermore, our statistical approach required the definition of acclimatization at the group level and thus depends on the sample size. Hence, inferences derived from subgroup analyses should be drawn with caution. When visually inspecting the acclimatization profiles (Fig. 2), it is apparent that certain parameters (step length, toe trajectory, ankle ROM, SSD hip/knee, SSD knee/ankle, stride time) may still be undergoing adaptation at the end of the 10-minute analysis. While none of these parameters met criteria for a steady unidirectional change (category 2), meaningful adaptation beyond 10 minutes cannot be ruled out. Experiments investigating longer-term adaptation are inevitably complicated by the onset of exhaustion, particularly in older and patient populations. Furthermore, longer acclimatization periods would have limited feasibility in research and clinical settings due to fatigue and time efficiency.

In this study, acclimatization was evaluated in a single session and subgroup analysis showed evidence of retention of certain aspects of acclimatization in those accustomed to treadmill walking. Clinical investigation of gait disorders is primarily focused on patient groups (multiple sclerosis, Parkinson’s disease), in whom acclimatization and its retention may be different. Further research is required to ascertain optimal acclimatization protocols in these important groups.

The norm data presented here for acclimatization to treadmill walking confirm the existence of considerable adaptations in a broad range of gait parameters describing many commonly-measured facets of human locomotion. This finding highlights the importance for the inclusion of acclimatization periods in clinical and research protocols using treadmill-based locomotor measures. The ideal duration of acclimatization will depend on the metric(s) under investigation, but most parameters studied here reached a steady state within 300–400 strides, usually equating to around 6–7 minutes at a comfortable walking speed. Age and treadmill naivety appear to affect the amplitude of acclimatization more than its duration, perhaps reflective of underlying motor learning modalities. Future research should focus on acclimatization patterns in clinically relevant patient groups. Standardization of acclimatization protocols for treadmill-based gait analysis will improve the quality of clinical examinations and trials using locomotor outcomes.

## Data Availability

All data generated or analysed in the framework of this study are included in this published article.
